# Predictors of cardiopulmonary fitness in cancer-affected and -unaffected women with a pathogenic germline variant in the genes *BRCA1/2 (*LIBRE-1)

**DOI:** 10.1038/s41598-022-06913-1

**Published:** 2022-02-21

**Authors:** A. Berling-Ernst, M. Yahiaoui-Doktor, M. Kiechle, C. Engel, J. Lammert, S. Grill, R. Dukatz, K. Rhiem, F. T. Baumann, S. C. Bischoff, N. Erickson, T. Schmidt, U. Niederberger, M. Siniatchkin, M. Halle

**Affiliations:** 1 Department of Prevention and Sports Medicine, School of Medicine, University Hospital Rechts der Isar, Technical University of Munich (TUM), Munich, Germany; 2 Institute for Medical Informatics, Statistics and Epidemiology (IMISE), University of Leipzig, Leipzig, Germany; 3 Department of Gynecology and Center for Hereditary Breast and Ovarian Cancer, University Hospital Rechts der Isar, Technical University of Munich (TUM), Munich, Germany; 4 Center for Familial Breast and Ovarian Cancer, Center for Integrated Oncology (CIO), University Hospital Cologne, Cologne, Nordrhein-Westfalen, Germany; 5 Institute of Nutritional Medicine, University of Hohenheim, Stuttgart, Germany; 6 Comprehensive Cancer Center Ludwig Maximillian University (CCC LMU), University of Munich Clinic, Campus Großhadern, Munich, Germany; 7 University Cancer Center Schleswig-Holstein (UCCSH), Kiel, Germany; 8 Institute of Medical Psychology and Medical Sociology, University Medical Center Schleswig Holstein, Kiel, Germany; 9 DZHK (German Centre for Cardiovascular Research), Munich, Germany

**Keywords:** Cancer, Psychology, Oncology, Risk factors

## Abstract

Physical activity (PA) helps prevention and aftercare of sporadic breast cancer (BC), cardiopulmonary fitness (CPF) being an age-independent predictor of tumor-specific mortality. Therefore, we wanted to identify predictors of CPF (represented by peak oxygen uptake: VO_2peak_) in *BRCA1/2* mutation carriers whose risk of developing BC is high. We used cross-sectional data from 68 *BRCA1/2* germline mutation carrying women participating in the randomized, prospective, controlled clinical study LIBRE-1. Assessments included cardiopulmonary exercise testing, medical and lifestyle history plus socioeconomic status. Additionally, the participants completed a psychological questionnaire regarding their attitude, subjective norms, perceived behavior control and intention towards PA. A multivariate logistic regression model was used to identify predictors for participants reaching their age- and sex-adjusted VO_2peak_ reference values. 22 participants (median age: 40 years, interquartile range (IQR) 33–46) were cancer-unaffected and 46 cancer-affected (median age: 44 years, IQR 35–50). The strongest predictor for reaching the reference VO_2peak_ value was attitude towards PA (Odds Ratio 3.0; 95% Confidence Interval 1.3–8.4; p = 0.021). None of the other predictors showed a significant association. A positive attitude towards PA seems to be associated with VO_2peak_, which should be considered in developing therapeutic and preventive strategies.

Trial registrations: NCT02087592; DRKS00005736.

## Introduction

The risk for cancer in women with a germline pathogenic variant of *BRCA1/2* is substantially elevated compared to the general population. For *BRCA1* mutation carriers, the risk estimate for breast cancer by age 80 is 72% and 44% for ovarian cancer. For *BRCA2* mutation carriers, the lifetime risk estimate for breast cancer is 69% and for ovarian cancer 26%^1^.

### Cardiopulmonary fitness (CPF)

In addition to the high risk of developing cancer, there is also increasing evidence that *BRCA1/2* mutation carriers are more likely to suffer from cardiovascular diseases^2^. Initial studies indicate, that VO_2peak_ could be a predictor of overall mortality^3^ and breast cancer specific mortality^4^ in sporadic breast cancer patients. Cardiopulmonary fitness, measured by peak oxygen consumption (VO_2peak_), is an assessment of cardiovascular function. A recent investigation found an inverse association between VO_2peak_ and several biomarkers linked to tumorigenesis^5^. A reduced VO_2peak_ favors tumor-related side effects, e.g. cancer related fatigue or reduced quality of life^6^. A high level of physical activity leads to a higher VO_2peak_ and thus better cardiopulmonary fitness. In a meta-analysis involving 571 cancer patients, a supervised exercise training was associated with significant improvements in VO_2peak_^7^.

Therefore, strategies to prevent or restore a weak VO_2peak_ in the large and fast-growing population of cancer survivors are of great clinical relevance. Hence, we wanted to identify factors, which influence physical activity behavior and consequently the VO_2peak_. Another two components are relevant in this undertaking, which are explained below.

### Physical activity in youth

Studies indicate that the high risk of cancer in *BRCA1/2* germline mutation carriers may be mitigated by physical activity, especially during adolescence and early adulthood^8,9^. In a retrospective, longitudinal case–control study of 886 *BRCA1/2* mutation carriers, Lammert et al. (2018) showed that women moderately active between the ages of 12–17 years had a 38% lower risk of developing premenopausal breast cancer compared to less active women^10^. A further retrospective cohort study (725 *BRCA1/2* mutation carriers) reported a 42% risk reduction for developing breast cancer among participants with increasing levels of sports activity prior to, but not after, age 30^11^.

### Theory of planned behavior (TPB)

The theory of planned behavior (TPB) includes the determinants attitude, subjective norm, perceived behavioral control and intention, and is validated for predicting physical activity behavior in breast cancer survivors. Studies have shown an association between attitude, the subjective norm, perceived behavioral control, and intention with regard to the current physical activity behavior in breast cancer survivors^12–14^.

We wanted to examine the association of the aforementioned factors (physical activity in youth and TPB) in particular, adjusted for other available co-factors in this study with VO_2peak_ in this high-risk group of *BRCA1/2* mutation carriers.

## Methods

The LIBRE-1 study (Lifestyle Intervention study in women with hereditary BREast and ovarian Cancer, 1 = pilot) is a multi-centric, prospective, randomized and controlled clinical trial. It is registered in the German Study Register for Clinical Trials (DRKS No.: DRKS00005736), as well as the study registry of the National Institutes of Health (NCT No.: NCT02087592) on 14/03/2014. The study was conducted according to the requirements of national laws and ICH E6 Good Clinical Practice (GCP) of June 1996. The recommendations of the Declaration of Helsinki in its current version were followed as well as the German Federal Data Protection Act (BDSG). The ethics review board of the Klinikum Rechts der Isar of the Technical University of Munich has approved the study protocol (Reference 5686/13). All participating centers and all participants provided written informed consent and the study design and methods are reported in detail elsewhere^15^.

### Study population

In the LIBRE-1 study, 68 cancer-unaffected or previously breast and/or ovarian cancer-affected women with a *BRCA1/2* germline mutation were recruited from three centers of the German Consortium for Hereditary Breast and Ovarian Cancer (GC-HBOC, www.konsortium-familiaerer-brustkrebs.de). Inclusion criteria were: pathogenic germline variant in the *BRCA1* or *BRCA2* gene, age ≥ 18 years and written informed consent. Exclusion criteria were: presence of metastatic tumor disease, life expectancy < 3 years, Body Mass Index (BMI) < 15 kg/m^2^, clinically limiting cardiopulmonary disease and Karnovsky-Index (describes the general condition of a patient) < 60%, blood pressure at rest > 160/100 mmHg. Further exclusion criteria were significant orthopedic or psychological problems that would not allow the participant to partake in a group intervention, or a current pregnancy^15^.

### Assessment of cardiopulmonary fitness

Cardiopulmonary fitness was determined by the peak oxygen uptake (VO_2peak)_ and assessed by cardiopulmonary exercise testing (CPET). The CPET was a ramp protocol (3 min sitting on the bicycle, 3 min steady state at 30 watts, continuous individual increase in wattage with the aim of achieving a maximal workload on the test-person within 8 to 12 min, 5 min recovery after exercise) with the target of exhausting them with a respiratory exchange ratio (RER) > 1.05. During the CPET, the participants were asked about their received perception of exertion (RPE) based on the Borg scale every 2 min. The Borg scale ranges between 6 (no exertion at all) to 20 (maximal exertion)^16^. The VO_2peak_ indicates the maximal number of milliliters of oxygen the body can utilize per minute when under workload. The VO_2peak_ can be used as a criterion for evaluating a person's physical endurance. In the present analysis, the aim was to determine which participants could reach their VO_2peak_ reference value. The VO_2peak_ reference value was calculated with the SHIP study formula (Study of Health in Pomerania). This formula considers the gender, age, height and weight of the participant in calculating their VO_2peak_ reference value^17^. In addition to the VO_2peak_, the determination of the oxygen uptake (VO_2_) at the first ventilatory threshold (VT1) as a criterion for the aerobic capacity was also assessed^18^.

### Questionnaire assessment of physical activity in youth, the attitude and rating towards physical activity (TPB) and the socioeconomic status

The physical activity in youth was determined with a clinical baseline interview using an international questionnaire, clinical baseline questionnaire. Participants’ activity ages 10 to 19 years were categorized into two groups: (1) Much less active and somewhat less active than their peers were classified as *Inactive in youth* and (2) participants who indicated that they were exactly as active or more active than their peers were classified as *Active in youth*.

The questionnaire for the assessment of the rating towards physical activity was developed based on the theory of the planned behavior of Icek Ajzen^19^. The TPB is validated for predicting physical activity behavior in breast cancer survivors^20,21^. The TPB assumes that positive attitudes such as *"exercise is fun"* and subjective norms "*my family thinks I should exercise more*", as well as perceived behavior control "*to exercise regularly is for me very feasible*", influence intentions. Intentions such as "*I intend to exercise regularly*” are assumed to be mediators of the influence of subjective norms and attitudes on behavior. TPB's summary statement is that people intend to behave, if they rate it positively, believe that important other individuals feel that they should do it, and that they perceive it to be under their own control^19^. Participants completed a psychological questionnaire “*Rating physical activity and nutrition*” (Bewertung körperlicher Aktivität und Ernährung = BKAE) based on the TPB. The 9 sections of the exercise-related part of the BKAE questionnaire include 44 questions on a 7-step scale and were standardized for the analysis so that the 4 TPB determinant scores (attitude, subjective norms, perceived behavior control and intention) would be comparable.

In the clinical baseline questionnaire the socioeconomic status was also determined^22^. For the analysis we used an adaptation of the Winkler-Stolzenberg Index (WSI = ((education-1)/6) + employed − smoker) based on the following items with values between 0 and 1.3^23^:

o whether the participant was currently employed
o whether the participant was a smoker
o the participant’s educational level (including school education and vocational training)

### Statistical analysis

**Figure 1:**
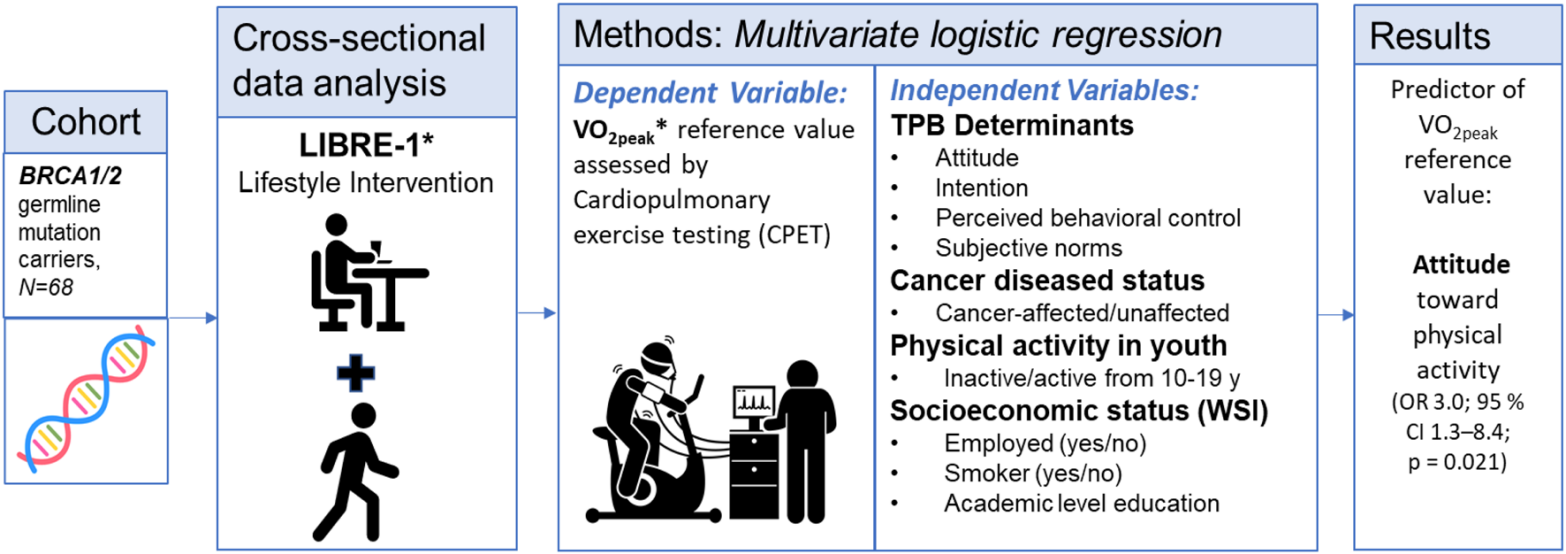
Graphical abstract of the analysis. *LIBRE-1 Lifestyle Intervention study in women with hereditary BREast and ovarian Cancer, 1 = pilot; *RCT* randomized controlled trial; *TPB* theory of planned behavior, *WSI* Winkler-Stolzenberg-Index; *VO_*2peak*_ reference values of the maximal oxygen uptake, *OR* odds ratio, *CI* confidence interval; *y* years. Pictures: miri019/shutterstock.com; leremy/shutterstock.com.

First all data were examined graphically and descriptively. T-Tests were carried out to compare the groups of cancer-unaffected and cancer-affected participants. Then, in order to determine possible parameters that influence the VO_2peak_ performance in the study sample, a multivariate logistic regression was carried out, where the dependent variable was the dichotomized VO_2peak_ (i.e. whether the participant reached at least their VO_2peak_ reference value, which is explained in detail above in the section “Assessment of cardiopulmonary fitness”^17^). The independent variables were the TPB determinants (standardized continuous score values from the BKAE questionnaire as explained in detail above), cancer-affected status (binary), physical activity in youth (binary) and the socioeconomic status’ value (as explained in detail above). In order to illustrate the aims and methods of this analysis, we have added a graphical abstract (Fig. 1).

All statistical analyses were carried out using R version 3.4.2 (R Core Team, 2017. R: A language and environment for statistical computing. R Foundation for Statistical Computing, Vienna, Austria. URL https://www.r-project.org). For all analyses, an alpha level of 0.05 was used to determine statistical significance (two-tailed).

### Ethical standards

The study was conducted according to the requirements of national laws and ICH E6 Good Clinical Practice (GCP) of June 1996. The recommendations of the Declaration of Helsinki in its current version were followed. The LIBRE-1 study protocol has been reviewed and approved by the ethics committees of the three participating centers (Munich: Reference No. 5686/13, Cologne: Reference No. 13-053 and Kiel: Reference No. B-235/13).

## Results

### Participant characteristics

Of the 68 participants, 46 (68%) were breast and/or ovarian cancer-affected of whom 44 had suffered from breast cancer and two from ovarian cancer, with one suffering from both. Of these 44 breast cancer-affected participants, 4 also indicated that they had had other cancers. Median time since the primary tumor diagnosis in this group was 3 years with a range of 0—23. The median age of the cancer-affected women at study entry was 44 years (IQR 35–50) compared to 40 years (IQR 33–46) in the other group. Further detailed comparisons and statistical testing between the two groups can be found in the publication by Kiechle et al. describing the first results from the LIBRE-1 study^24^.

Results from the CPET showed a median VO_2peak_ of 26 ml/min/kg in both groups, while median VO_2_ at VT1 as its percentage was 61.9% in the cancer-affected group and 60.9% in the cancer-unaffected group (Table 1). Of the cancer-affected participants, 28 (61%) considered themselves as active as their peers during their youth in comparison to 19 (86%) of the cancer-unaffected participants.

**Table 1:** Characteristics of the participants.

	Cancer-affected participants (n = 46)	Cancer-unaffected participants (n = 22)	Total (n = 68)
Age (years)	44 (35–50)	40 (33–46)	42 (33–50)
Smoker/former smoker, n (%)	34 (74%)	10 (48%)	44 (65%)
In employment, n (%)	31 (67%)	20 (91%)	51 (75%)
Academic level education, n (%)	19 (41%)	13 (59%)	32 (47%)
Winkler-Stolzenberg Index*	0.33 (0.0–1.0)	1.0 (1.0–1.3)	1.0 (0.33–1.33)
Physically active in youth, n (%)	28 (61%)	19 (86%)	47 (69%)
Body mass index (kg/m^2^)	23.0 (21.0–26.6)	25.9 (22.1–32.7)	23.2 (21.3–27.6)
Height (cm)	168.0 (166.0–172.3)	166.5 (163.8–171.5)	168.0 (164.8–172.3)
Weight (kg)	65.7 (60.0–75.3)	69.9 (60.6–81.5)	67.7 (60.0–76.2)
VO_2peak_ (ml/min/kg)	26.0 (21.5–31.0)	26.0 (22.0–30.0)	26.0 (21.5–31.0)
VO_2_ at VT1 as percentage of VO_2peak_ (%)	61.9 (55.5–68.6%)	60.9 (56.8–63.6%)	61.3 (56.1–68.1%)
BORG (RPE/RPD)	18.0 (18.0–19.0)	19.0 (18.0–19.0)	18.0 (18.0–19.0)
RER (VCO_2_/VO_2_)	1.19 (1.15–1.23)	1.17 (1.11–1.23)	1.18 (1.14–1.23)
Attitude	0.30 (− 0.22 to 0.65)	0.04 (− 0.83 to 0.21)	0.13 (− 0.31 to 0.47)
Intention	− 0.63 (− 0.84 to 0.39)	0.39 (− 0.43 to 0.80)	− 0.43 (− 0.84 to 0.39)
Perceived behavior control	− 0.55 (− 0.93 to 0.19)	0.50 (− 0.36 to 1.16)	− 0.17 (− 0.74 to 0.83)
Subjective norms	0.01 (− 0.68 to 0.76)	− 0.37 (− 0.91 to 0.17)	− 0.04 (− 0.72 to 0.61)

Data are presented as median (interquartile range); Active in youth = those who indicated that they were exactly as active or more active than their peers from ages 10 to 19 years; *BMI* Body Mass Index; *VO*_*2peak*_ Maximal Oxygen Uptake; *VT1* ventilatory threshold one; *RER* respiratory exchange ratio; *RPE* rate of perceived exertion/*RPD* rate of perceived dispnoe.

*Adaptation of the WSI to describes the socioeconomic status (compare method part).

Cancer-unaffected participants had lower standardized median attitude and subjective norms scores in comparison to cancer-affected participants (attitude: 0.04 versus (vs) 0.30, subjective norms: − 0.37 vs 0.01), while their median intention and perceived behavior control scores were higher compared to cancer-affected group (intention: 0.39 vs − 0.63, perceived behavior control: 0.50 vs − 0.55) (Table 1).

### Multivariate logistic regression on reaching VO_2peak_ reference values

As illustrated in Table 2, participants with higher attitude scores showed significantly higher odds of reaching the VO_2peak_ reference values (Odds ratio (OR) 3.0; 95% confidence interval (95% CI) 1.3–8.4; p = 0.021). The intention (OR 1.9; 95% CI 0.7–5.3; p = 0.192) scores showed a trend for higher chances of reaching the VO_2peak_ reference values, whereas perceived behavior control (OR 0.4; 95% CI 0.2–1.0; p = 0.067) and subjective norms (OR 0.8; 95% CI 0.4–1.5; p = 0.491) scores showed the opposite tendency.

**Table 2:** Logistic regression results for predicting the VO_2peak_ reference value.

Parameter	Odds ratio	95% Confidence interval	p-value
Attitude	3.0	1.3–8.4	**0.021**
Intention	2.0	0.7–5.3	0.192
Perceived behavior control	0.4	0.2–1.0	0.067
Subjective norms	0.8	0.4–1.5	0.491
Cancer disease status	1.8	0.4–7.9	0.438
Active in youth	0.6	0.2–2.1	0.398
Socioeconomic status (WSI)	1.4	0.7–2.9	0.417

Active in youth = binary variable indicating whether they were exactly as active or more active than their peers from ages 10 to 19 years.

*WSI* Winkler-Stolzenberg Index to describe the socioeconomic status.

Cancer-unaffected participants and those with a higher socioeconomic status had higher odds of reaching their VO_2peak_ reference values (OR = 1.8; CI 0.4–7.9; p = 0.438 and OR = 1.4; CI 0.7–2.9; p = 0.417 respectively). Those who indicated they were active in their youth had lower odds of reaching their VO_2peak_ reference values (OR = 0.6; CI 0.2–2.1; p = 0.398).

## Discussion

The purpose of this analysis was to identify predictors of cardiopulmonary fitness (VO_2peak)_ in cancer-unaffected and cancer-affected *BRCA1/2* mutation carriers.

Of all determinants, a positive attitude towards physical activity (a TPB component) was the only significant predictor found in our analysis (OR 3.0; 95% CI 1.3–8.4; p = 0.021) for reaching the participant’s VO_2peak_ reference value. A previous meta-review across 72 studies suggests that generally people’s attitudes, seem to be the key influences in forming intentions to participate in physical activity^25^. According to the TPB it is necessary to have an intention, such as the intention of *“walking half an hour every day”* to carry out the intended behavior in accordance with the attitudes, subjective norms, and perceived behavioral control. Courneya et al. established that attitude and subjective norm were significant determinants of intention in a study of 164 sporadic breast cancer patients, and intention and perceived behavioral control in turn were significant factors of physical activity behavior during breast cancer treatment^20^.

In our analysis, the intention, subjective norms, and perceived behavioral control played a subordinate role for reaching the VO_2peak_ reference value, which may indicates that attitude towards physical activity should be considered when implementing physical activity programs, which consequently influences the cardiopulmonary fitness. A qualitative study by Smith et al. reported that participants' attitudes toward physical activity were negatively influenced by receiving little information from the oncologist and health professionals. A lack of social support and structured exercise programs were also identified as barriers to physical activity implementation^26^. Therefore, treatment strategies for *BRCA1/2* mutation carriers should contain information dissemination and education about the beneficial effects of physical activity before and after tumor occurrence to improve the participants’ attitude towards physical activity. In addition, cancer patients should be educated that physical activity is feasible and safe during tumor therapy. For this, it can be advantageous to include, inform and educate people around the participant accordingly.

It has been shown that age, sex, health status, self-efficacy and motivation are associated with higher levels of physical activity. Baumann et al. described that the personal attitude towards exercise and physical activity behavior in youth and adolescence and the intention to maintain a physical activity behavior are important components, which influence current physical activity^27^. Therefore, we included physical activity in youth in our analysis, however no association was found. It should be noted that the activity in youth was only surveyed retrospectively in our analysis and did not include any information on the duration and intensity of the respective physical activity.

VO_2peak_ is determined by genetics, gender, body composition, physical activity behavior and age^28^. The VO_2peak_ decreases by about 8–10% per decade from the 30th year of life^29^. In cancer patients, VO_2peak_ has been found to be about 30% lower compared to healthy peers^7^. The reference values of VO_2peak_ were age-adjusted so that the age-related decrease in cardiopulmonary fitness was implicitly included in our analysis. We found no significant association between cancer status and reaching the VO_2peak_ reference value in our study.

We had expected that previously cancer-affected *BRCA1/2* mutation carriers would reach their VO_2peak_ reference value less often compared to the cancer-unaffected participants. Usually, there are great differences between cancer-unaffected and previously cancer-affected women depending on the respective tumor therapy^29,30^. Various tumor therapies in combination with a sedentary lifestyle additionally led to marked impairments in cardiopulmonary fitness and may not recover after treatment^31,32^. This circumstance has not yet been investigated in *BRCA1/2* mutation carriers. However, some studies have shown that the knowledge of an increased cancer risk or a positive *BRCA* genetic test induces lifestyle changes^33–35^. Positive lifestyle changes, which promote a higher amount of physical activity can lead to a better cardiopulmonary fitness. This could be even more important especially after a cancer diagnosis and might explain our results.

For sporadic breast cancer patients, the TPB might be a viable framework to promote physical activity successfully during cancer treatment. However, the salient beliefs of breast cancer patients concerning physical activity were different from those of the cancer-unaffected population^20^. Whether this also applies to cancer-affected *BRCA1/2* mutation carriers and has an influence on the VO_2peak_ still needs to be investigated. If these findings are validated, the attitude should be taken into account in the treatment and preventive measures for *BRCA1/2* mutation carriers with regard to cancer risk and prognosis.

### Limitations

Physical activity in youth and adolescence was recorded retrospectively and only in comparison with the peer group. This meant that no quantitative information was available on the extent, duration, intensity and type of physical activity. In future, this information should also be collected in order to ensure better comparability and significance with regard to physical activity in youth.

As our results come from a feasibility study it should be noted that the informative value is limited by the small sample size and must be interpreted accordingly.

## Conclusion

In conclusion, a positive attitude towards physical activity seems to play a role in the current VO_2peak_ status in cancer-unaffected and cancer-affected *BRCA1/2* mutation carriers. Physical activity during adolescence and the health and socioeconomic status did not influence VO_2peak_ levels. These relationships need to be investigated in a larger sample size, for instance in the ongoing LIBRE-2 study. If these results are verified, future concepts and studies should consider the attitude towards being physically active in managing *BRCA1/2* mutation carriers.
